# Genome-wide analysis and identification of stress-responsive genes of the CCCH zinc finger family in *Capsicum annuum* L.

**DOI:** 10.3389/fpls.2023.1189038

**Published:** 2023-05-30

**Authors:** Wenchen Tang, Yupeng Hao, Xinyu Ma, Yiqi Shi, Yongmeng Dang, Zeyu Dong, Yongyan Zhao, Tianlun Zhao, Shuijin Zhu, Zhiyuan Zhang, Fenglin Gu, Ziji Liu, Jinhong Chen

**Affiliations:** ^1^Hainan Institute, Zhejiang University, Sanya, China; ^2^Spice and Beverage Research Institute, Sanya Research Institute, Chinese Academy of Tropical Agricultural Sciences/Hainan Key Laboratory for Biosafety Monitoring and Molecular Breeding in Off-Season Reproduction Regions, Sanya, China; ^3^Tropical Crops Genetic Resources Institute, Chinese Academy of Tropical Agricultural Sciences/Key Laboratory of Crop Gene Resources and Germplasm Enhancement in Southern China, Ministry of Agriculture, Haikou, China; ^4^College of Agriculture and Biotechnology, Zhejiang University, Hangzhou, China

**Keywords:** gene family, CCCH, phylogenetic analysis, *Capsicum annuum*, stress

## Abstract

The CCCH zinc finger gene family encodes a class of proteins that can bind to both DNA and RNA, and an increasing number of studies have demonstrated that the CCCH gene family plays a key role in growth and development and responses to environmental stress. Here, we identified 57 CCCH genes in the pepper (*Capsicum annuum* L.) genome and explored the evolution and function of the CCCH gene family in *C. annuum*. Substantial variation was observed in the structure of these CCCH genes, and the number of exons ranged from one to fourteen. Analysis of gene duplication events revealed that segmental duplication was the main driver of gene expansion in the CCCH gene family in pepper. We found that the expression of CCCH genes was significantly up-regulated during the response to biotic and abiotic stress, especially cold and heat stress, indicating that CCCH genes play key roles in stress responses. Our results provide new information on CCCH genes in pepper and will aid future studies of the evolution, inheritance, and function of CCCH zinc finger genes in pepper.

## Introduction

Zinc finger proteins (ZFPs), which are named for their ability to bind zinc to form a stable finger-like structure, are sequence-specific transcription factors that usually contain varying numbers of cysteine (Cys) and histidine (His) residues. Cys and His are used to chelate zinc ions to form a zinc finger structure, which can recognize and bind to DNA (Hall, 2005). Zinc finger proteins are also associated with the metabolism of different types of RNAs in organisms (Hall, 2005) and can specifically bind to DNA, RNA, and DNA–RNA complexes to regulate gene expression. Several gene families have been identified in plants based on their function and structure, including the RING finger (Freemont, 1993; Kosarev et al., 2002), CCCH (Li et al., 2001), DOF (Lijavetzky et al., 2003), WRKY (Zhang and Wang, 2005), ERF (Nakano et al., 2006), and LIM (Arnaud et al., 2007) families. Zinc finger protein motifs can be divided into different types according to the number of conserved Cys and His residues and the spacing between these residues, such as C2H2, C8, C6, C3HC4, C2HC5, C4, C4HC3, and CCCH (Berg and Shi, 1996; Takatsuji, 1998; Moore and Ullman, 2003; Schumann et al., 2007). CCCH zinc finger proteins generally contain at least one zinc finger motif. Three Cys and one His residue are the most important components of this motif. The common sequence of the CCCH motif can be defined as C-X_4-15_-C-X_4-6_C-X_3-4_-H (where X stands for any amino acid, numbers indicate the number of amino acids, C is Cys, and H is His), and C-X_7-8_-C-X_5_-C-X_3_-H is the largest sequence among CCCH proteins (Wang et al., 2008).

CCCH zinc finger proteins are involved in plant development, adaptation, hormonal regulation, and the regulation of processes related to physiological adversity, especially responses to biotic and abiotic stress. In *Arabidopsis*, *AtTZF1*, which consists of two zinc finger motifs separated by 18 amino acids, is a CCCH-type zinc finger protein (Iuchi and Kuldell, 2005). Overexpression of *AtTZF1* enhances the tolerance of *Arabidopsis thaliana* to cold and drought stress and affects the growth and stress responses mediated by abscisic acid (ABA) and gibberellic acid (GA) (Lin et al., 2011). The expression patterns of *AtTZF1*, *AtTZF2*, and *AtTZF3* are similar (Lee et al., 2012). *AtC3H49*/*AtTZF3* and *AtC3H20*/*AtTZF2* can regulate growth rate, plant size, leaf and flower morphology, as well as aging and lifespan. Overexpression of these two genes can attenuate transpiration, enhance drought tolerance, alter growth patterns, and delay senescence (Lee et al., 2012). In addition, the CCCH zinc finger proteins HUA1 and HUA2 play a role in AGAMOUS pre-mRNA processing and in floral reproductive organ identity (Li et al., 2001; Cheng et al., 2003). In rice, OsTZF1 improves stress tolerance by regulating the RNA metabolism of stress-responsive genes (Jan et al., 2013). GhZFP1 in cotton contains two typical zinc finger motifs (C-X_8_-C-X_5_-C-X_3_-H and C-X_5_-C-X_4_-C-X_3_-H) that improve drought and disease resistance in transgenic tobacco (Guo et al., 2009). The overexpression of *GmZF351* in transgenic soybeans activates lipid biosynthesis genes, accelerates the accumulation of seed oil, and thus increases the seed oil content (Li et al., 2017). In cucumber, *CsSEF1* encodes protein containing three conserved zinc finger motifs, two of which are CCCH motifs. The expression of *CsSEF1* is up-regulated in leaves and flowers; it plays a role in later developmental stages after embryogenesis and the signal transduction pathway of fruits from photoassimilate limitation to the sink organs (Grabowska et al., 2009; Tazuke and Asayama, 2013). In pepper, the CCCH zinc finger protein CaC3H14 regulates antagonistic interactions between salicylic acid (SA) and jasmonic acid (JA)/ethylene (ET) signaling, which enhances the resistance of plants to *Ralstonia solanacearum* infection (Qiu et al., 2018).

A total of 68, 67, 68, 91, 34, 62, 80, 89, 103, 116, 31, and 86 CCCH zinc finger family genes have been identified in *Arabidopsis* (Wang et al., 2008), rice (Wang et al., 2008), maize (Peng et al., 2012), poplar (Chai et al., 2012), *Medicago truncatula* (Zhang et al., 2013), citrus (Liu et al., 2014), tomato (Xu, 2014), banana (Mazumdar et al., 2017), cabbage (*Brassica rapa*) (Pi et al., 2018), soybean (Hu and Zuo, 2021), rose (Li et al., 2021), and tobacco (Tang C. et al., 2022), respectively. Although CCCH zinc finger proteins play an important role in many aspects of plant growth and development, no systematic studies have been conducted to analyze and identify members of the CCCH gene family in pepper to date.

Pepper has the highest vitamin C content among all vegetables, which can promote appetite and improve digestion. Whole-genome sequencing and bioinformatics analysis can be used to identify and analyze CCCH zinc finger genes involved in the growth and development, metabolism, and adaptation to stress in pepper plants (Kim et al., 2014; Qin et al., 2014). Here, we identified 57 CCCH zinc finger genes in the pepper genome. We also systematically analyzed the phylogenetic structure, domains, conserved motifs, chromosome localization, duplication events, collinearity, and tissue-specific expression patterns of these CCCH zinc finger genes, and this provided insights into the roles of CCCH gene family members in the growth and development of pepper plants. Finally, the published RNA sequencing (RNA-seq) data were used to investigate the expression of CCCH genes in different tissues, such as the roots, stems, and leaves, and the expression patterns of the genes were validated using quantitative real-time polymerase chain reaction (qRT-PCR). These results provide new insights that will aid future studies of the functions of candidate genes involved in the growth, development, adaptation, hormone regulation, and stress physiology of pepper plants.

## Materials and methods

### Identification and characterization of CCCH zinc finger family members in pepper

In this study, we used genomic data from *Capsicum annuum* cv. CM334. First, we downloaded amino acid sequences for all *Capsicum* proteins from the Phytozome database^1^ (Tuskan et al., 2006; Goodstein et al., 2012) and amino acid sequences for CCCH (PF00642, Zinc finger C-X_8_-C-X_5_-C-X_3_-H type, and similar sequences) from the Pfam database^2^ (El-Gebali et al., 2019). The CCCH motif was used to retrieve the amino acid sequence of peppers in hmmsearch^3^ with a threshold of E-value < 1 × 10^-5^. All the obtained protein sequences were submitted to the Pfam database and SMART domain search database^3^ to confirm the structural integrity of the zf_CCCH domain. Furthermore, we made use of the Pfam^2^ and SMART^4^ databases to clarify the structural integrity of the ZF_CCCH domain (Schultz et al., 2000). We extracted sequences of the conserved domains from the identified pepper CCCH proteins. We used the ExPASy tool^5^ (Gasteiger et al., 2005) to calculate the number of amino acids, isoelectric point (pI), molecular weight (Mw), and other physical and chemical properties of the zinc finger CCCH protein sequences.

### Classification and sequence analysis of the CCCH genes

We downloaded amino acid sequences for pepper, tomato, and rice from the Phytozome database^1^. *Arabidopsis* CCCH zinc finger genes were identified from the *Arabidopsis* information resource website^6^. Sequences were aligned using the neighbor-joining method, and the evolutionary tree was constructed in MEGA 11 software (Kumar et al., 2018). Branch support was tested by performing 1,000 bootstrap replications. The phylogenetic tree was uploaded in Newick format to the EvolView web server^7^ to visualize the tree. The subfamily classification of the *Capsicum* CCCH gene family was based on a previously published classification for *Arabidopsis thaliana* (Wang et al., 2008). MCScanX^8^ was used to characterize syntenic relationships among *CCCH* genes in *Arabidopsis*, tomato, and pepper.

### Gene structure and conserved motif analysis

We downloaded genome sequences and coding sequences from the Phytozome database^1^ to analyze the structure of CCCH gene family members. The structure of the CCCH genes was plotted using TBtools (Chen et al., 2020). MEME Suite Version 5.4.1^9^ was used to identify the conserved motifs of CCCH gene family members in pepper, with the maximum motif search number set to 10, and other parameters set to their default values. Any repetitions were considered a motif position that was distributed throughout the sequence (Bailey et al., 2009).

### Chromosome location and collinearity analysis

Detailed chromosomal mapping was obtained from GFF genomic files downloaded from the Phytozome database^1^ to visualize the chromosomal distribution of the CCCH genes in pepper in TBtools (Chen et al., 2020). We also identified tandem duplication events in CCCH family genes using MCScanX in TBtools. MCScanX in TBtools and BLASTP searches were used to identify the segmental duplication events of CCCH genes in pepper and clarify collinearity relationships between genes in different species (Wang et al., 2012; Chen et al., 2020). The non-synonymous (Ka) and synonymous (Ks) substitutions between gene pairs was calculated by using TBtools.

### Analysis of CCCH gene expression by RNA-seq under different conditions

We analyzed the expression profiles of pepper CCCH zinc finger genes in different tissues, under different types of biotic stress and abiotic stress, and in the presence of different phytohormones by downloading the following RNA-seq datasets from the National Center for Biotechnology Information (NCBI) Gene Expression Omnibus^10^: flower, root, stem, placenta, and pericarp (stage 1, 2, and 3) of pepper plants during the mature green (MG) stage, breaker (B) stage, and 5 and 10 days after the breaker stage (BioProject ID: PRJNA223222); 30 min, 4 h, 1 day, 2 days, and 3 days after infection with PepMoV and TMV (BioProject ID: PRJNA223222); 1, 3, 6, 12, and 24 h under cold, heat, drought, and salt stress (BioProject ID: PRJNA525913); and 1, 3, 6, 12, and 24 h after MeJA, SA, ET, and ABA treatment (BioProject ID. PRJNA634831) (Kim et al., 2014; Kang et al., 2020; Lee et al., 2020). The fragments per kilobase of exon model per million mapped reads (FPKM) values were calculated using Hisat2 (v2.0.5) and Sringtie (v2.1.7) software with the following formula: log(FPKM+1). These data were then visualized using the ‘pheatmap’ package in R software.

### Stress treatments and collection of materials

In this experiment, gene expression levels of CCCH genes were detected using the pepper cultivar CM334. All pepper plants were sown and grown under greenhouse conditions (16 h light/8 h dark, 25-28°C). When peppers had six true leaves, the experimental groups were subjected to cold treatment (16 h light/8 h dark, 10°C) and heat treatment (16 h light/8 h dark, 40°C) in the incubator, and the leaves were collected at 0, 3, 6, 12, 24, and 72 h after the treatment. Three replicates were collected from three different plants, immediately frozen in liquid nitrogen, and then stored in a -80°C refrigerator.

### qRT-PCR verification

The RNA sample was extracted using an RNAprep Pure Plant Plus Kit (Tiangen) according to the manufacturer’s instructions. The DNAase-treated RNA was reverse-transcribed with M-MLV (RNase H-) reverse transcriptase. qRT-PCR was performed using a CFX96TM Real-Time system (Applied Biosystems). Primers (20-24 bp) were designed using the Primer-BLAST tool in NCBI, and the amplicon lengths were 80-220 bp (**Supplementary Table 1**). All settings were set to their default values. Three technical replicates were performed for each gene, and *UBI3* was used as the internal reference gene. The total volume of each reaction was 20 µL, which consisted of 2 µL of cDNA, 1 µL of gene-specific primers, 7 µL of ddH_2_O, and 10 µL of 2× ChamQ Universal SYBR qPCR Master Mix reagent. The thermal cycling conditions were as follows: 95°C for 10 min, followed by 40 cycles at 95°C for 15 s and 60°C for 1 min. At the end of the cycle, a solubility-free curve was generated to analyze the expression of each gene tested.

## Results

### Identification and characterization of CCCH transcription factor family members in pepper

In this study, 57 CCCH genes were identified from the *C. annuum* cv. CM334 genome using the Hidden Markov Model of LEA against the genome database of *C. annuum*. These CCCH genes were renamed from *PEPTY1* to *PEPTY57* according to their order on chromosome 1-12 (**Supplementary Table 2**). All identified CCCH genes encoded proteins ranging from 295 to 1015 amino acids, and their predicted isoelectric points (pI) ranged from 4.7 to 9.39. To investigate the sequence characteristics of the most common CCCH motifs in the pepper CCCH zinc finger proteins, we extracted amino acid sequences from CCCH conserved regions (Thompson et al., 1997). The CCCH domain mainly consisted of a triple cysteine and a histidine, and the following motif was commonly observed (C-X_7-8_-C-X_5_-C-X_3_-H) (**Supplementary Figure 1**).

### Phylogenetic tree and sequence structure analysis

**Figure 1:**
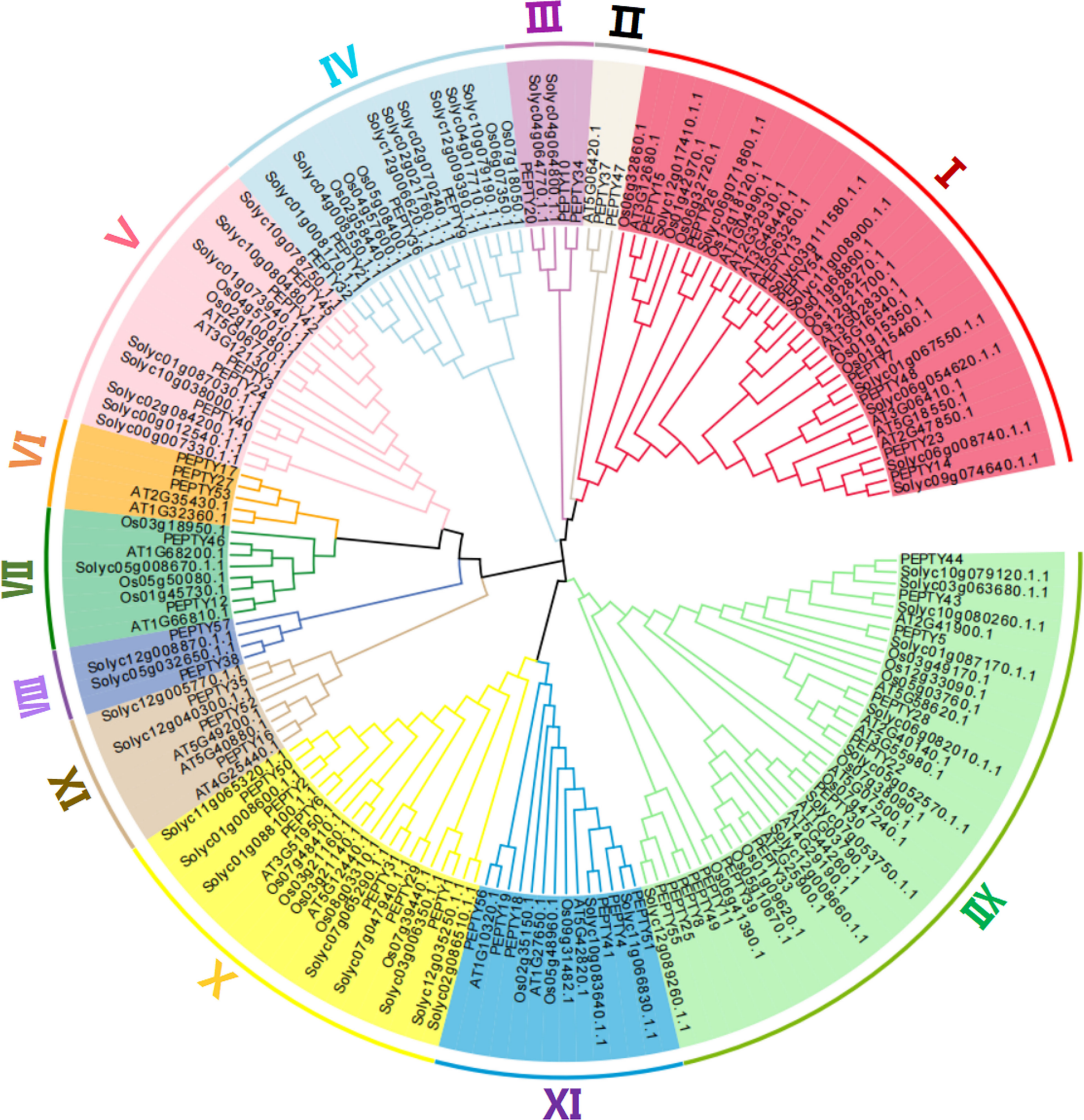
Evolutionary tree of CCCH genes in *Arabidopsis thaliana*, *Oryza sativa*, *Solanum lycopersicum*, and *Capsicum annuum*. The different shades of color correspond to different subgroups.

We constructed a phylogenetic tree using the entire amino acid sequence of each member of pepper, *Arabidopsis*, tomato, and rice to explore the evolutionary relationships among CCCH zinc finger genes. As shown in **Figure 1**, the pepper CCCH zinc finger genes were divided into 12 groups based on previous studies of *Arabidopsis*. The number of CCCH zinc finger genes in each group was uneven. Group XII was the largest (13 CCCH zinc finger genes), followed by Group I (8 CCCH zinc finger genes) and Group II, VII, and VIII (each with 2 CCCH zinc finger genes). Group III, IV, V, VI, IX, X, and XI have 3, 4, 5, 3, 3, 6, and 6 CCCH zinc finger genes, respectively.

**Figure 2:**
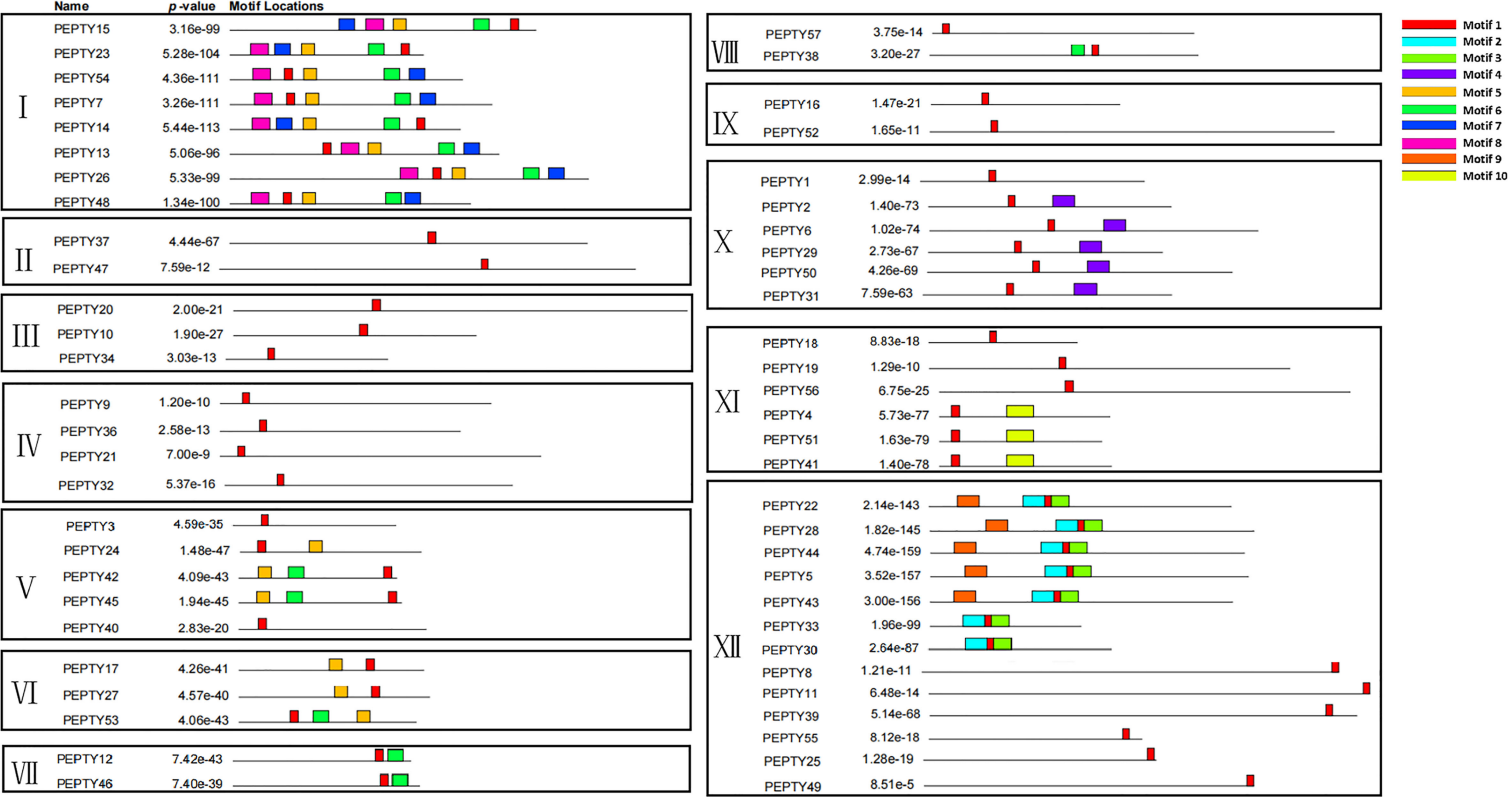
Protein motifs of the CCCH gene family in pepper. The colorful boxes delineate different motifs. The clustering was performed according to the results of the phylogenetic analysis.

We performed a structure analysis of the 57 CCCH zinc finger genes in pepper. All the CCCH genes had introns and exons, but they varied greatly in size and number. The number of exons ranged from 1 to 14 (**Supplementary Table 3**). Most of the genes had less than 10 exons. The average number of exons per gene was 5.4. Genes in Group VI and VII both had two exons, and genes in Group XII contained only one exon. However, genes in Group VIII had 10 exons. Subsequently, the conserved motifs of the CCCH genes in pepper were identified using the online MEME suite program. Ten conserved motifs were detected, ranging from 6 to 50 amino acids in length (**Figure 2**; **Supplementary Table 3**). Unsurprisingly, the structure of the genes in the same subclade was similar. The five conserved motifs 1, 5, 6, 7, and 8, were all found in Group I. Motif 5, 7, and 8 had the C-X_8_-C-X_5_-C-X_3_-H structure. Motif 4 was only present in Group X, motif 5 was widely present in Group V and VI, motif 9 was only present in Group XII, and motif 10 (C-X_7_-C-X_5_-C-X_3_-H) was only present in Group XI. Most genes in the same branch had similar conserved motif compositions and structures, which suggests that they were functionally similar.

### Chromosomal locations and duplications of CCCH zinc finger genes in pepper

**Figure 3:**
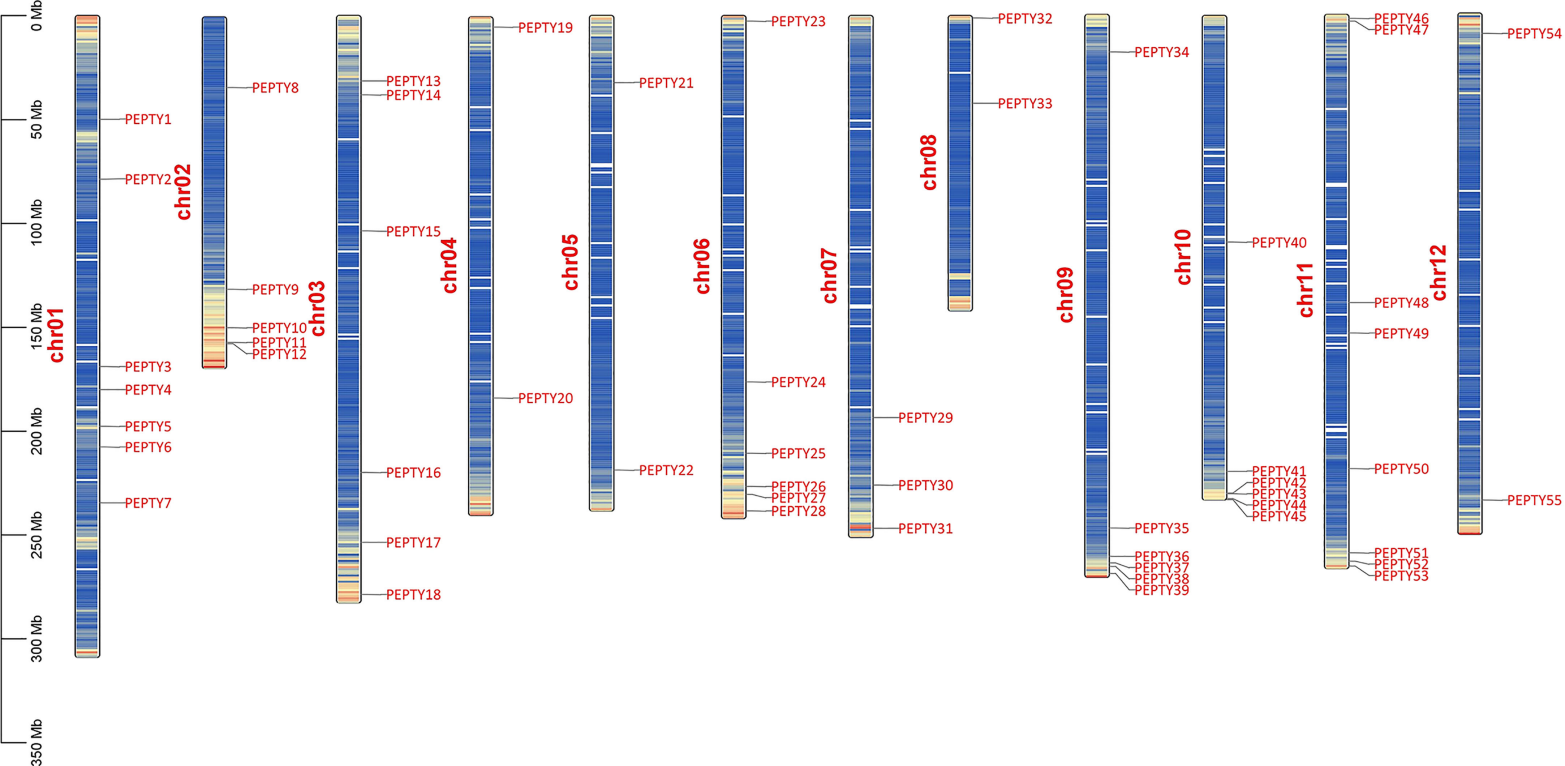
Chromosomal distribution of CCCH genes in pepper. Chr01–12 indicate chromosomes 01–12. Bands on the chromosomes indicate gene density.

Using the pepper genome annotation information and TBtools (Tuskan et al., 2006; Chen et al., 2020), we characterized the chromosomal distribution of CCCH zinc finger genes. A total of 55 of the 57 CCCH zinc finger genes identified could be mapped on chromosomes; *PEPTY56* and *PEPTY57* were the two genes that could not be mapped. As shown in **Figure 3**, these 55 CCCH genes were unevenly distributed across the 12 chromosomes, and the number of genes on each chromosome was not related to chromosome size. For example, the largest chromosome (Chr 01) contained seven CCCH genes; however, the chromosome containing the most genes was Chr 11, which had eight CCCH genes. Chr 05 and 12 had only two CCCH genes, which was the same number of CCCH genes contained on the shortest chromosome (Chr 08).

**Figure 4:**
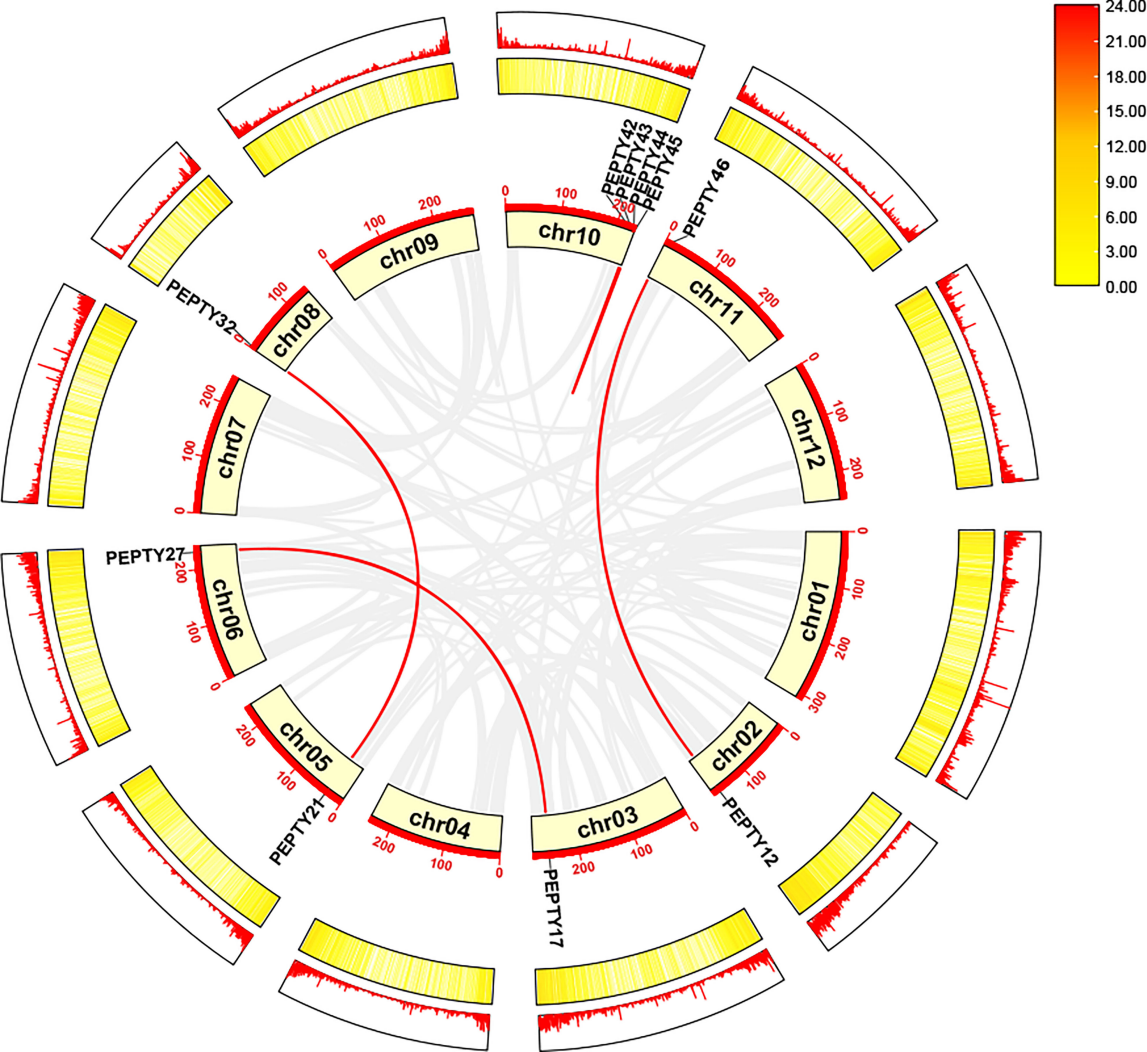
Collinearity analysis of the CCCH gene family in pepper. Chromosomes 01–12 are represented by yellow rectangles. The gray lines indicate syntenic blocks in the pepper genome, and the red lines between chromosomes delineate segmentally duplicated gene pairs.The outermost heatmap and lines represent gene density on the chromosomes.

Next, we identified tandem duplication events using the Multiple Collinearity Scan toolkit (MCScanX) in TBtools. No tandem duplication events were identified. Thus, we identified segmental duplication events using MCScanX in TBtools and BLASTP searches (Wang et al., 2012; Chen et al., 2020). A total of 5 segmentally duplicated gene pairs were detected, and these were detected across nine chromosomes (**Figure 4**). On chromosomes 10, 2 pairs of genes (*PEPTY42/PEPTY45* and *PEPTY43*/*PEPTY44*) on the same chromosomes appear to be products of segmental duplication events. Segmental duplication events were not detected on Chr 01, 04, 07, 09, and 12. These findings indicate that segmental duplication events appear to have played a key role in shaping the diversity of CCCH genes in pepper.

We also investigated collinearity relationships between pepper CCCH genes and associated genes from *Arabidopsis* and *Solanum lycopersicum* to identify homologous genes. Collinearity relationships were observed between 14 pepper genes and 20 *Arabidopsis* genes and between 40 pepper genes and 42 tomato genes. A total of 21 pairs of homologous genes were identified between pepper and *Arabidopsis*, and 47 pairs of homologous genes were identified between pepper and tomato (**Supplementary Figure 3**). The logarithm of homologous genes with tomato was twice that of homologous genes with *Arabidopsis*; and this is likely because the closer phylogenetic relationship between pepper and tomato (both in the family Solanaceae) than between pepper and *Arabidopsis*. To assess the selective constraint pressure of gene pairs, Ka/Ks calculations were performed in TBtools (**Supplementary Table 4**). Most gene pairs have Ka/Ks ratios below 1, indicating that purification selection may have been undertaken during evolution.

### Expression analysis of *PEPTY* genes in different pepper tissues

**Figure 5:**
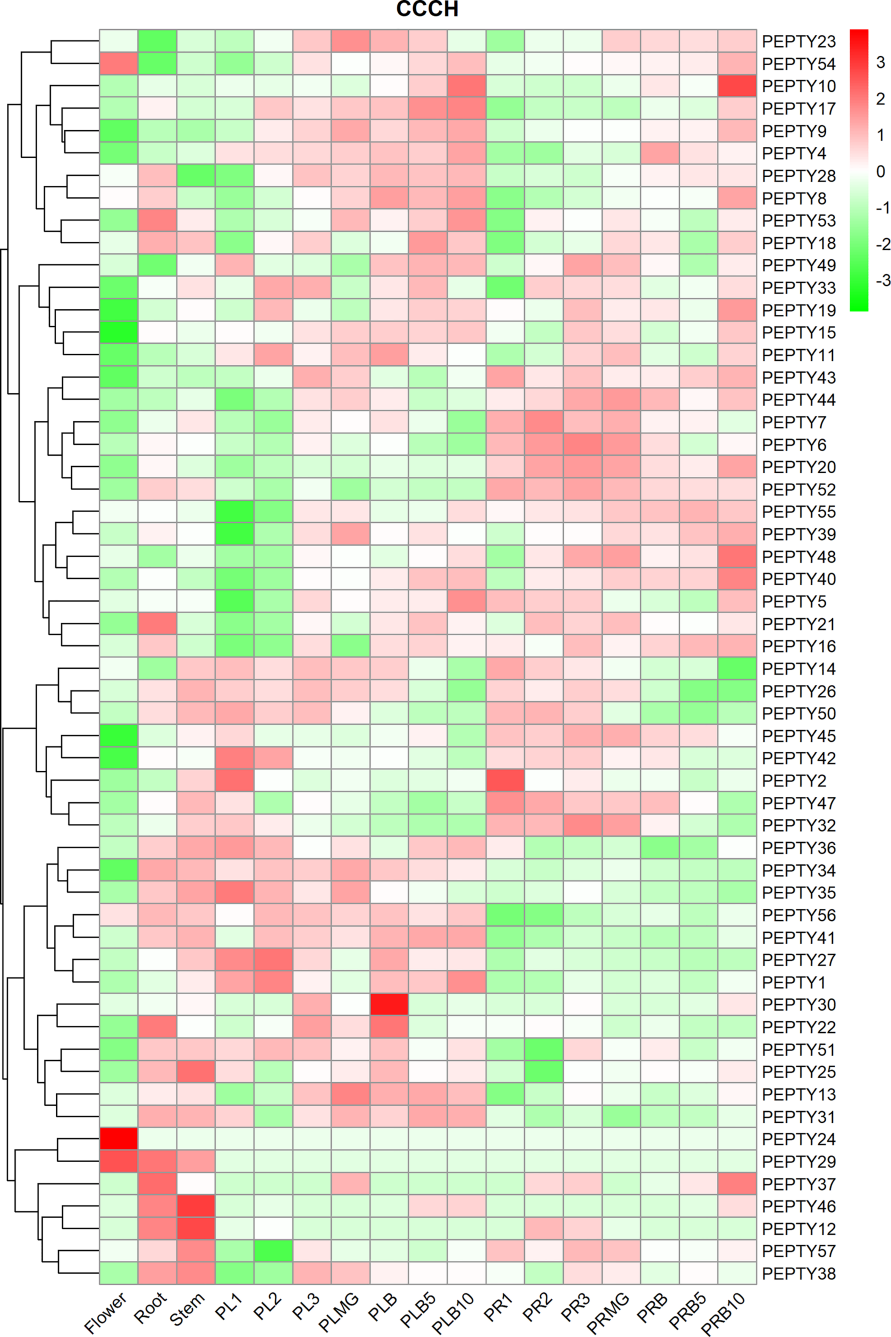
Hierarchical clustering of expression profiles of pepper CCCH genes in different organs. The heatmap was constructed using the ‘pheatmap’ package in R software, and the fragments per kilobase of exon model per million mapped reads (FPKM) values of the CCCH genes were converted to log(FPKM+1) values. The different tissues included flower, root, stem, placenta (PL), and pericarp (PR). MG denotes mature green, and B denotes breaker. 1, 2, and 3 indicate stage. 5 and 10 indicate days. Red indicates a high relative abundance of transcripts. Green indicates a low relative abundance of transcripts.

We characterized the expression of pepper CCCH genes in five tissues: flower, root, stem, placenta, and pericarp tissue (**Figure 5**; **Supplementary Table 5**). *PEPTY24* was expressed at high levels in flowers and at low levels in the roots and stems; *PEPTY12* and *PEPTY46* were expressed at high levels in stems, but their expression gradually decreased in the roots and flowers as development advanced. *PEPTY29* was most highly expressed in the flowers, followed by the roots and stems. In placenta period, the expression of *PEPTY10* gradually increased with developmental stage. The expression of *PEPTY30* was the highest in the initial breaker stage. The expression of *PEPTY35* was up-regulated at the early developmental stage in the placenta and was down-regulated at the breaker stage. In pericarp period, the expression of *PEPTY10* was significantly up-regulated at day 10 of the breaker stage. The expression of *PEPTY2* was high at stage 1 in both the placenta and pericarp period (PL1 and PR1) and decreased thereafter. The expression of CCCH might vary among organs and at different growth and developmental stages. Some of these genes such as *PEPTY24* and *PEPTY30* are likely involved in the growth and development of pepper.

### Expression analysis of *PEPTY* genes under different stress conditions and phytohormone treatments

**Figure 6:**
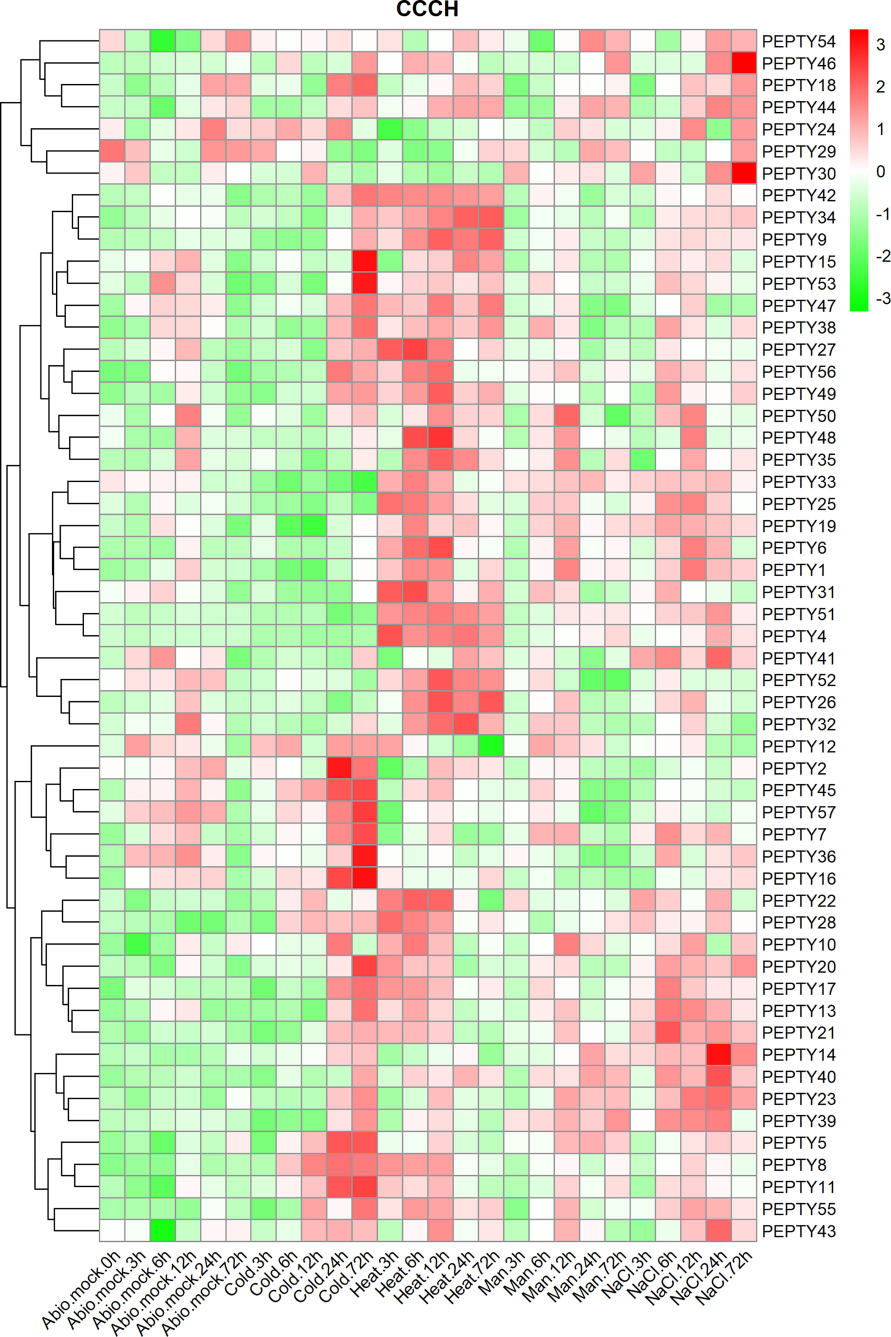
Expression profiles of pepper CCCH genes under different types of abiotic stress. Abiotic stresses included cold, heat, drought (D-mannitol), and salt (NaCl). Time points include 1, 3, 6, 12, and 24 h. The control group is indicated by Abio.mock labels. Red indicates a high relative abundance of transcripts. Green indicates a low relative abundance of transcripts.

Analysis of the relative transcript abundance of *PEPTY* genes under different types of abiotic stress revealed that the expression of many of these genes was significantly up-regulated under cold, heat, drought (D-mannitol) and salt (sodium chloride, NaCl) stress (**Figure 6**; **Supplementary Table 5**). The expression of *PEPTY2, PEPTY5, PEPTY7, PEPTY8, PEPTY11, PEPTY16, PEPTY36, PEPTY45*, and *PEPTY57* was up-regulated under cold stress. The expression of *PEPTY4, PEPTY9, PEPTY26, PEPTY32, PEPTY34, PEPTY42, PEPTY51*, and *PEPTY52* was significantly up-regulated at all time points under heat stress. The expression of *PEPTY6, PEPTY31, PEPTY32*, and *PEPTY48* was highest at 12, 6, 24, and 12 h, respectively. By contrast, the expression of *PEPTY14, PEPTY30, PEPTY40*, and *PEPTY46* was up-regulated at 24, 72, 24, and 72 h, respectively, under salt stress. Under drought stress, the expression of *PEPTY5, PEPTY10, PEPTY14, PEPTY23, PEPTY39*, and *PEPTY40* was up-regulated.

**Figure 7:**
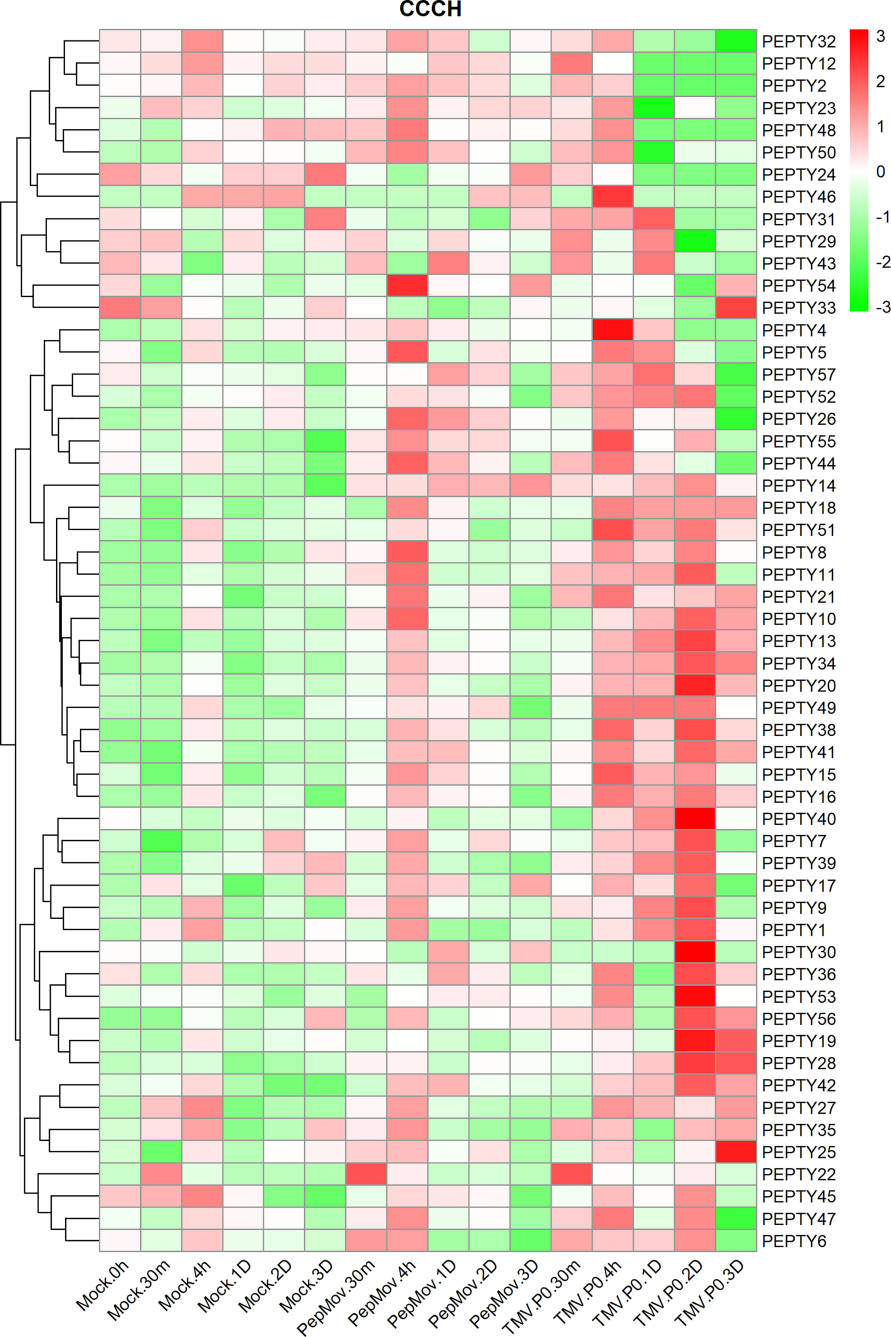
Expression profiles of pepper CCCH genes under different types of biotic stress. Biotic stresses included pepper mottle virus (PepMoV) and tobacco mosaic virus (TMV). Time points include 30 min, 4 h, 1 d, 2 d, and 3 d. The control group is indicated by mock labels. Red indicates high relative abundance of transcripts. Green indicates low relative abundance of transcripts.

The expression of CCCH genes after treatment with two viruses was performed to clarify their responses to biotic stress (**Figure 7**; **Supplementary Table 5**). The expression of *PEPTY22* following pepper mottle virus (PepMoV) treatment was highest 30 min post-treatment and decreased thereafter. The expression of most genes, such as *PEPTY8, PEPTY11*, and *PEPTY54*, was up-regulated 4 h post-treatment. By contrast, the expression of *PEPTY22* was significantly up-regulated 30 min after treatment with tobacco mosaic virus (TMV), which was consistent with its response to PepMoV treatment. The expression of *PEPTY4* and *PEPTY46* was high 4 h after TMV treatment. In addition, the expression of *PEPTY20, PEPTY28, PEPTY30, PEPTY40*, and *PEPTY53* was high 2 days after TMV treatment. The expression of *PEPTY25* and *PEPTY33* was high 3 days after TMV treatment. The responses of most CCCH genes were more pronounced to TMV treatment than to PepMoV treatment.

**Figure 8:**
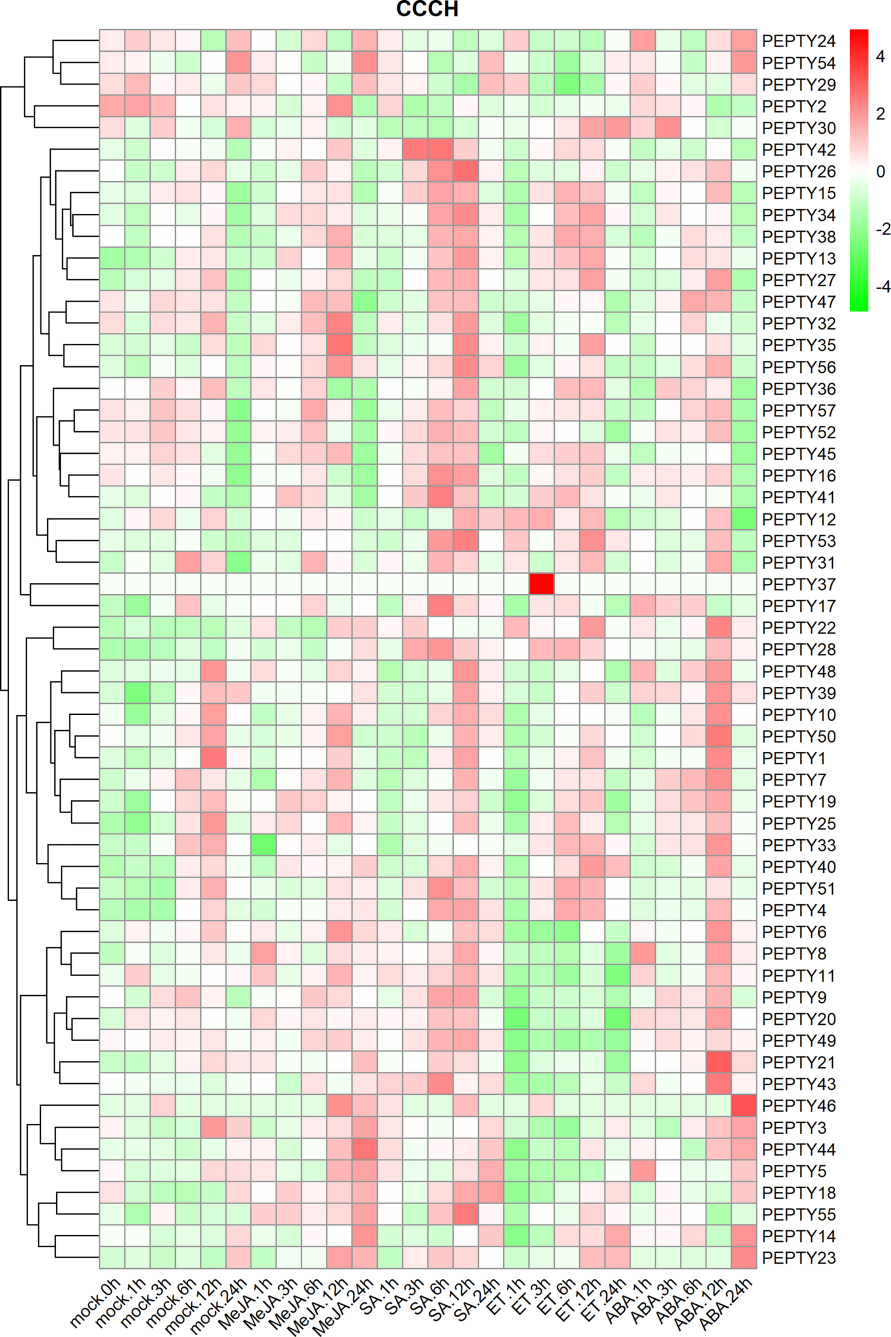
Expression profiles of pepper CCCH genes under phytohormone treatments. The phytohormone treatments included methyl jasmonate (MeJA), salicylic acid (SA), ethylene (ET), and abscisic acid (ABA). Time points include 1, 3, 6, 12, and 24 h. The control group is indicated by a mock label. Red indicates a high relative abundance of transcripts. Green indicates a low relative abundance of transcripts.

Ultimately, the expression profiles of CCCH genes were further analyzed under treatment with four phytohormones. The results are shown in **Figure 8**. The expression of *PEPTY8, PEPTY14, PEPTY22, PEPTY35, PEPTY44, PEPTY55*, and *PEPTY56* was increased after methyl jasmonate (MeJA) treatment. The expression of 13 genes (*PEPTY4, PEPTY13, PEPTY15, PEPTY26, PEPTY27, PEPTY28, PEPTY34, PEPTY35, PEPTY41, PEPTY42, PEPTY43, PEPTY53*, and *PEPTY56*) increased after SA treatment. The expression of *PEPTY35, PEPTY41, PEPTY42, PEPTY43, PEPTY53*, and *PEPTY56* was up-regulated after SA treatment. The expression of *PEPTY37* significantly increased 3 h after ET treatment. This gene was not expressed in the other treatments or the control. However, the expression of *PEPTY9, PEPTY11, PEPTY20, PEPTY21*, and *PEPTY49* was down-regulated. The expression of *PEPTY21* and *PEPTY43* was up-regulated after ABA treatment, especially at 12 h, and the expression of *PEPTY46* was more significantly up-regulated at 24 h. These results suggest that CCCH genes play a role in the response to phytohormones.

### qRT-PCR validation of the CCCH genes under cold and heat stress

**Figure 9:**
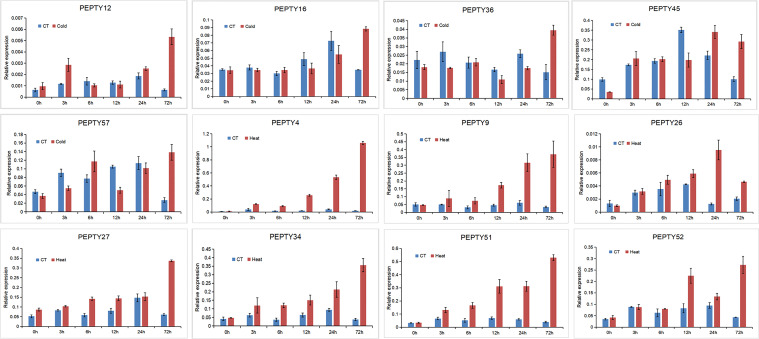
qRT-PCR analysis of 12 pepper CCCH genes under different stress treatments. The x-axis shows the time points after stress treatments. The y-axis shows the relative expression levels normalized to the reference gene *UBI3*. Data are mean ± SD of three technical replicates.

We conducted qRT-PCR analysis on 5 genes that were significantly up-regulated under cold treatment and 7 genes with expression patterns that varied under heat treatment in the heat map (**Figure 9**). Under cold stress, the expression of four genes (*PEPTY12, PEPTY16, PEPTY36*, and *PEPTY57*) peaked at 72 h, whereas the expression of *PEPTY45* peaked at 24 h. The expression of all these genes did not significantly differ from that of the control under cold treatment in the early stage; however, at 72 h, the expression of genes under cold treatment was at least two-fold higher than that of genes in the control group. A similar pattern was observed for *PEPTY4, PEPTY9, PEPTY26, PEPTY27, PEPTY34, PEPTY51*, and *PEPTY52* under heat treatment, and the significance of differences was even more pronounced. The expression of *EPTY4, PEPTY9, PEPTY27, PEPTY34, PEPTY51*, and *PEPTY52* peaked at 72 h, whereas the expression of *PEPTY26* peaked at 24 h. Differences in the expression of *PEPTY4, PEPTY9*, and *PEPTY51* between the heat treatment and control group gradually increased over time.

## Discussion

*C. annuum* is one of the most widely grown solanaceous vegetables worldwide and capsaicin produced from seed of *C. annuum* is an economically important spice, medicine, vegetable, and biopesticide. However, previous studies have shown that pepper plants are highly sensitive to biotic and abiotic stresses, such as pathogens, drought, cold, and heat (Kim et al., 2014; Kang et al., 2020; Lee et al., 2020). CCCH proteins have been identified in plants. These proteins are rather unusual in that they can regulate the expression of genes by binding to mRNA in addition to DNA (Kim et al., 2014; Qin et al., 2014). Functional analyses of CCCH genes in *Arabidopsis*, rice, maize, poplar, alfalfa (*Medicago truncatula*), citrus, tomato, banana, cabbage, soybean, rose, tobacco, and other plants have been conducted (Wang et al., 2008; Chai et al., 2012; Peng et al., 2012; Zhang et al., 2013; Liu et al., 2014; Xu, 2014; Mazumdar et al., 2017; Pi et al., 2018; Hu and Zuo, 2021; Li et al., 2021; Tang C. et al., 2022).

We identified 57 CCCH zinc finger genes in the genome of *C. annuum* cv. CM334. A total of 80 CCCH genes have been identified in tomato belonging to (Xu, 2014), which is also a member of the family Solanaceae. We searched for CCCH genes in the *C. annuum* L. Zunla-1 genome. However, this species only had 69 CCCH genes (**Supplementary Table 6**), which was lower than in tomato. The CCCH genes in CM334 could be divided into 12 subfamilies, and Group III and VIII genes were only present in pepper and tomato, but not in *Arabidopsis thaliana* and rice (**Figure 1**). These subfamilies are likely unique to the Solanaceae family.

Structural analysis of the CCCH genes revealed that the CCCH motifs are highly conserved, motif type and motif position were highly similar within each subfamily, but motif type and motif position varied among most subfamilies. The similarity and specificity within and between subfamilies, respectively, indicated that genes in the same subfamily may have similar functions, and genes in different subclades may perform different functions. No motifs in *PEPTY*35 were in Group IX, and 56.1% of pepper CCCH genes had at least two motifs. The main structures present were C-X_5_-C-X_4_-C-X_3_-H and C-X_7-8_-C-X_5_-C-X_3_-H.

Gene duplication is one of the primary drivers of the evolution of genomic and genetic systems. Duplicated genes have the potential to develop new functions. Gene family expansion in the genome generally stems from tandem and segmental duplication events (Moore and Purugganan, 2003; Cannon et al., 2004; Levasseur and Pontarotti, 2011). In Group V, there are five pepper CCCH genes (*PEPTY3, PEPTY24, PEPTY40, PEPTY42*, and *PEPTY45*), but only two *Arabidopsis* CCCH genes (*AtC3H36* and *AtC3H52*) and two rice CCCH genes (*OsC3H14* and *OsC3H31*). Two homologs of *Arabidopsis* or rice were likely generated by segmental duplication, and the pepper CCCH genes likely underwent one round of whole-genome duplication and one tandem duplication.

The expression levels of CCCH genes in pepper varied significantly among tissues and developmental stages (Chai et al., 2012; Li et al., 2021). Only the expression of *PEPTY24, PEPTY29*, and *PEPTY54* was up-regulated in flowers. The expression of *PEPTY24* was specific to flowers, which may be involved in the regulation of flowering in pepper. *PEPTY29* was expressed in flower, root, and stem, but not in placenta and pericarp; this gene might thus be involved in regulating flower, root, and stem development. Twenty-five genes were expressed in the roots, and 27 genes were expressed in the stems. The expression patterns of CCCH genes in pepper differ from those of CCCH genes in *Arabidopsis* and rice, where most CCCH genes are expressed in the roots, inflorescences, leaves, and seeds (Wang et al., 2008).

The expression profiles of CCCH genes under biotic stress, abiotic stress, and phytohormone treatments showed that most *PEPTY* genes were highly expressed under these conditions. Comparison with other studies confirmed that the activity of most CCCH zinc finger proteins can be induced by hormones such as ABA and GA; they may play a role in hormone-mediated signaling pathways (Verma et al., 2016; Han et al., 2021). This pattern of activity is similar to that observed under biotic and abiotic stress; it is even likely that a particular gene could respond to multiple different treatments. For example, in rice, the *OsTZF1* gene responds to GA, MeJA, and salicylate (Jan et al., 2013). In *Arabidopsis*, the expression of *AtOZF1* was highly induced by ABA and salinity treatment (Huang et al., 2011). High expression of *AtTZF2* and *AtTZF3* enhances tolerance to high salt stress, and the silencing of these two genes reduces the tolerance of plants to salt and drought stress (Huang et al., 2011; Huang et al., 2012; Lee et al., 2012). In addition, *AtTZF4, 5*, and *6* are positive regulators of ABA (Bogamuwa and Jang, 2013). These results enhance our understanding of the growth of pepper plants, as well as the response of pepper to various types of stress and hormone treatments.

After identifying CCCH genes in pepper that play significant roles in responses to cold and heat stress, the expression patterns of five candidate genes that were highly induced by cold stress and seven candidate genes that were highly induced by heat stress were validated by qRT-PCR. *PEPTY4* and *PEPTY51*, which were both in Group XI, were not expressed under cold stress and in the control environment, but they were highly expressed under heat stress. However, both *PEPTY16* and *PEPTY52* belonged to Group XI; the former was highly expressed under cold stress, and the latter was highly expressed under heat stress. *PEPTY36* in Group IV was highly expressed under cold treatment at 72 h. *PEPTY9*, which also belongs to the same subfamily as *PEPTY36*, was not significantly expressed under cold stress, but its expression was gradually up-regulated under heat stress. Thus, the expression patterns were not always the same among each subfamily member of each CCCH gene in pepper. One plausible explanation for this observation is that pepper is more sensitive to low-temperature and high-temperature stress. In addition, the responses of different genes to cold and heat might vary (Wang et al., 2019; Wang et al., 2021; Yang et al., 2021; Gao et al., 2022; Tang B. et al., 2022; Zhang et al., 2022). Therefore, further functional studies of these CCCH genes are needed to clarify the pathways underlying their responses to cold stress and heat stress.

## Conclusion

In this study, the phylogenetic relationships, structure, conserved motifs, chromosomal localization, duplication events, and expression profiles of CCCH genes were analyzed and 57 CCCH zinc finger genes were identified in pepper. A phylogenetic tree was constructed using CCCH sequences from *Arabidopsis*, tomato, and rice. Based on studies of *Arabidopsis*, we divided the pepper CCCH genes into 12 subfamilies. The exon/intron structure and motif composition were conserved in most subfamily. These genes were unevenly distributed on 12 chromosomes, and segmental duplication events appear to have been the major driver of gene expansion in the CCCH family. We characterized the expression profiles of CCCH genes in different tissues of pepper and under various types of stress and validated these expression patterns using qRT-PCR analysis. We found that CCCH zinc finger genes play important roles in biological processes such as growth and development and adaptation to stress. Overall, our findings will aid future studies aimed at examining the evolution, inheritance, and function of CCCH zinc finger genes in pepper and other plants.

## Data availability statement

The datasets presented in this study can be found in online repositories. The names of the repository/repositories and accession number(s) can be found in the article/**Supplementary Material**.

## Author contributions

JC, ZZ, SZ, TZ, ZL, FG and WT designed the research. WT, YZ, XM, YS and YD performed the research. WT, YH, and ZD analyzed the data. WT, ZZ, ZL, FG, and JC wrote the manuscript. All authors contributed to the article and approved the submitted version.

## Footnotes

1
https://phytozome-next.jgi.doe.gov

2
http://pfam.xfam.org/

3
http://www.hmmer.org/

4
http://smart.embl.de/smart/batch.pl

5
http://web.expasy.org/

6
https://www.arabidopsis.org/

7
http://www.evolgenius.info/evolview/

8
https://github.com/wyp1125/MCScanX

9
https://meme-suite.org/meme/index.html

10
https://www.ncbi.nlm.nih.gov/geo/query/acc.cgi
