# Supplementary material for: Genome-wide analysis and identification of stress-responsive genes of the CCCH zinc finger family in Capsicum annuum L
Source: Front Plant Sci. 2023 May 30;14:1189038. doi: 10.3389/fpls.2023.1189038 (PMC10262688; doi:10.3389/fpls.2023.1189038)
Supplement: Supplementary file 1 [file DataSheet_1.docx]

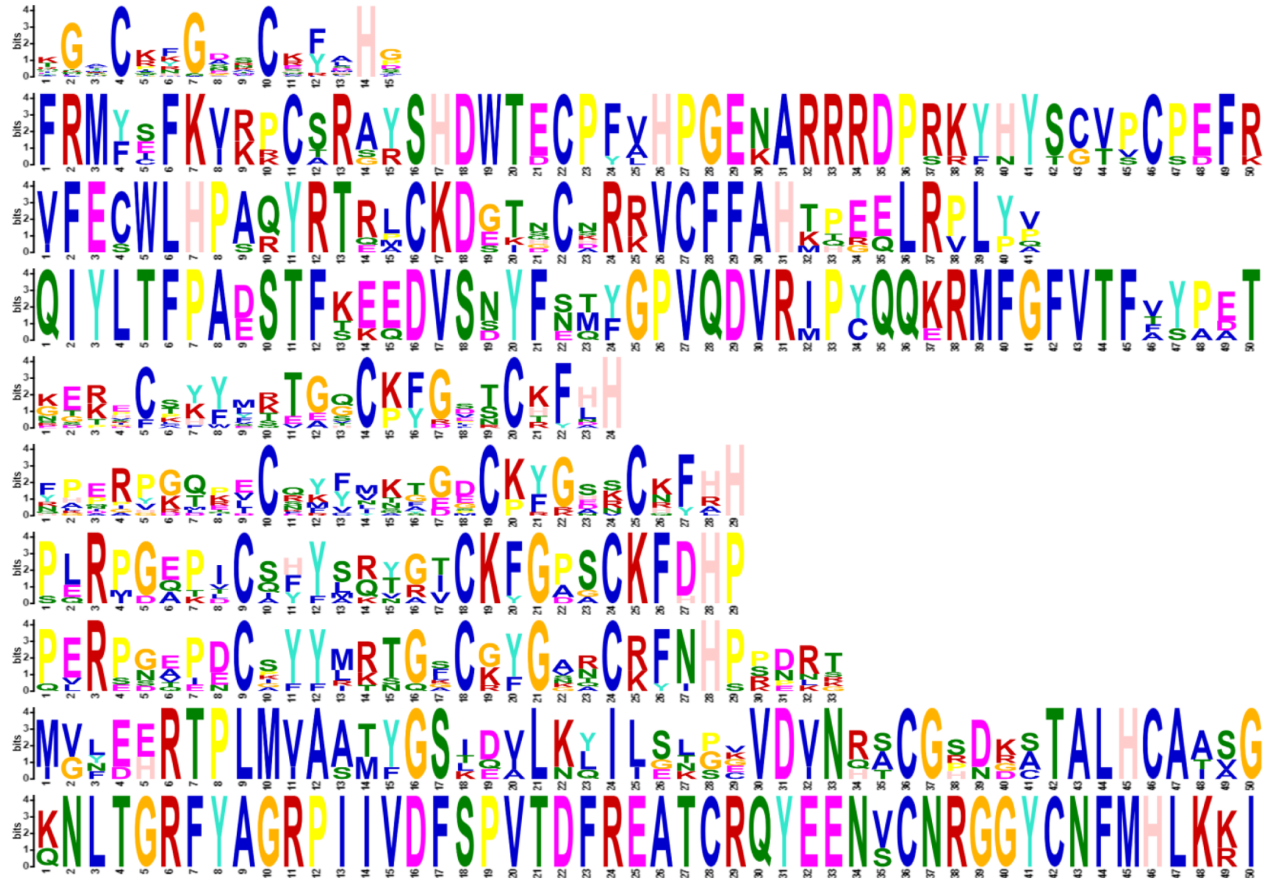


**Supplementary figure 1** The conserved motifs of CCCH zinc finger family. The total height of the letter piles at each position indicates the conservation of the sequence at that position (measured in bits). The height of a single letter in the letter piles represents the relative frequency of the corresponding amino acid at that position.


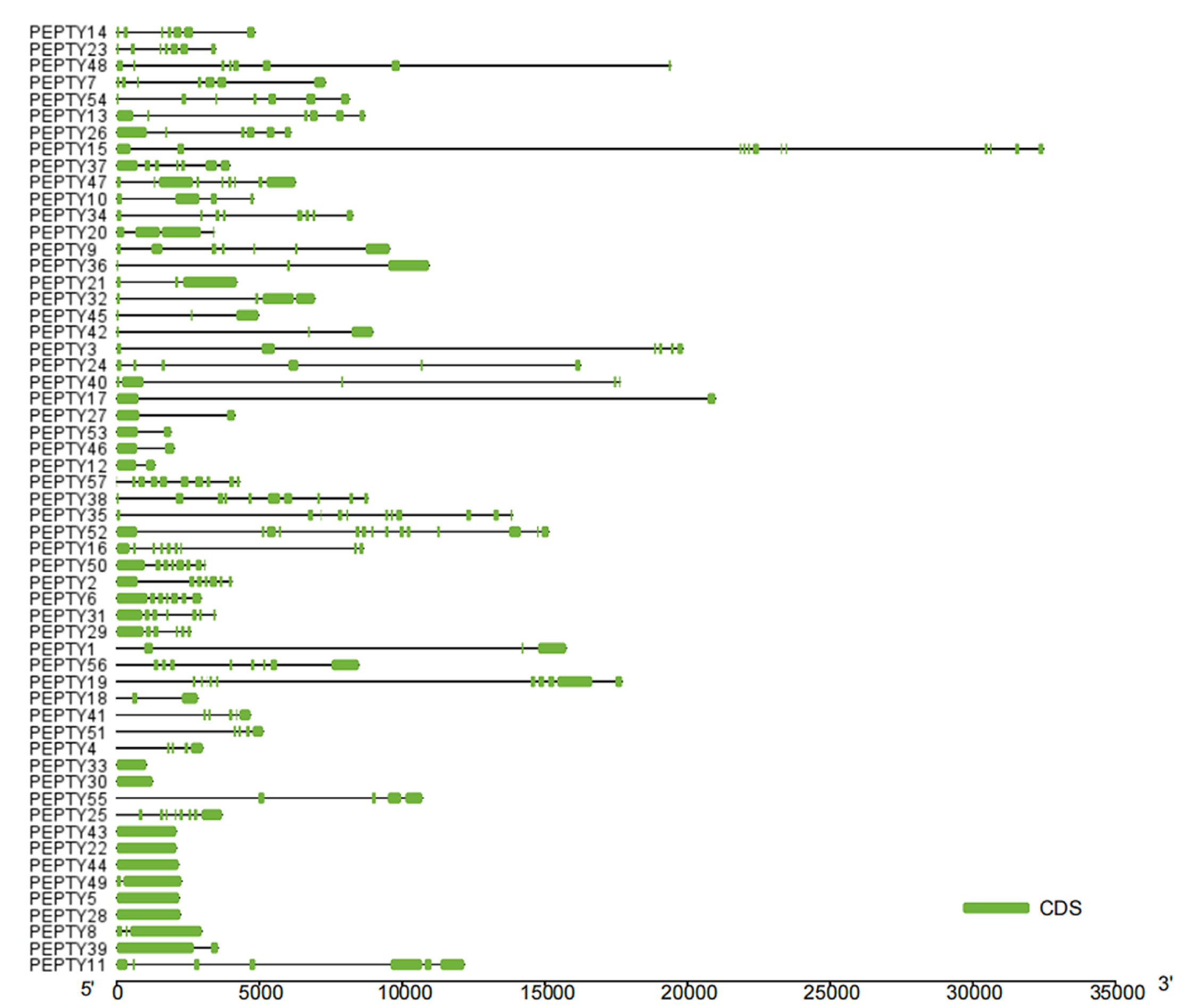


**Supplementary figure 2** Structure of CCCH genes in pepper. Exons and 5′-UTR/3′-UTR regions are shown using black lines and green bars, respectively. Black dotted lines denote introns.


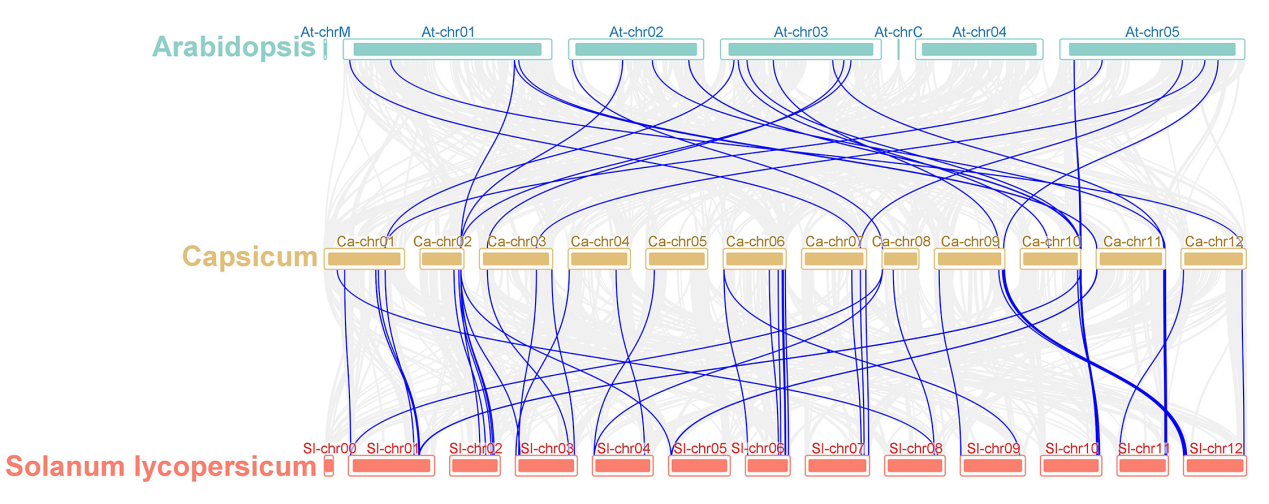


**Supplementary figure 3** Synteny analysis of CCCH genes between pepper and two other plant species. The gray lines indicate gene blocks in pepper that are orthologous to the other genomes. The blue lines delineate the syntenic CCCH gene pairs.
